# The failure of two major formaldehyde catabolism enzymes (ADH5 and ALDH2) leads to partial synthetic lethality in C57BL/6 mice

**DOI:** 10.1186/s41021-020-00160-4

**Published:** 2020-06-03

**Authors:** Jun Nakamura, Darcy W. Holley, Toshihiro Kawamoto, Scott J. Bultman

**Affiliations:** 1 Department of Environmental Sciences and Engineering, University of North Carolina at Chapel Hill, Chapel Hill, NC USA; 2 Laboratory of Laboratory Animal Science, Graduate School of Life and Environmental Biosciences, Osaka Prefecture University, Izumisano, Osaka, Japan; 3 Department of Genetics, University of North Carolina at Chapel Hill, Chapel Hill, NC USA; 4 Department of Environmental Health, University of Occupational and Environmental Health, Kitakyushu, Fukuoka, Japan

**Keywords:** ADH5, ALDH2, formaldehyde metabolism, Knockout mice, Synthetic lethality

## Abstract

**Background:**

Exogenous formaldehyde is classified by the IARC as a Category 1 known human carcinogen. Meanwhile, a significant amount of endogenous formaldehyde is produced in the human body; as such, formaldehyde-derived DNA and protein adducts have been detected in animals and humans in the absence of major exogenous formaldehyde exposure. However, the toxicological effects of endogenous formaldehyde on individuals with normal DNA damage repair functions are not well understood. In this study, we attempted to generate C57BL/6 mice deficient in both *Adh5* and *Aldh2*, which encode two major enzymes that metabolize endogenous formaldehyde, in order to understand the effects of endogenous formaldehyde on mice with normal DNA repair function.

**Results:**

Due to deficiencies in both ADH5 and ALDH2, few mice survived past post-natal day 21. In fact, the survival of pups within the first few days after birth was significantly decreased. Remarkably, two *Aldh2*^*−/−*^*/Adh5*^*−/−*^ mice survived for 25 days after birth, and we measured their total body weight and organ weights. The body weight of *Aldh2*^*−/−*^*/Adh5*^*−/−*^ mice decreased significantly by almost 37% compared to the *Aldh2*^*−/−*^*/Adh5*^*+/−*^ and *Aldh2*^*−/−*^*/Adh5*^*+/+*^ mice of the same litter. In addition, the absolute weight of each organ was also significantly reduced.

**Conclusion:**

Mice deficient in both formaldehyde-metabolizing enzymes ADH5 and ALDH2 were found to develop partial synthetic lethality and mortality shortly after birth. This phenotype may be due to the accumulation of endogenous formaldehyde. No serious phenotype has been reported in people with dysfunctional, dominant-negative ALDH2*2 alleles, but it has been reported that they may be highly susceptible to osteoporosis and neurodegenerative diseases. It is important to further investigate these diseases in individuals with ALDH2*2 alleles, including an association with decreased metabolism, and thus accumulation, of formaldehyde.

## Introduction

**Fig. 1:**
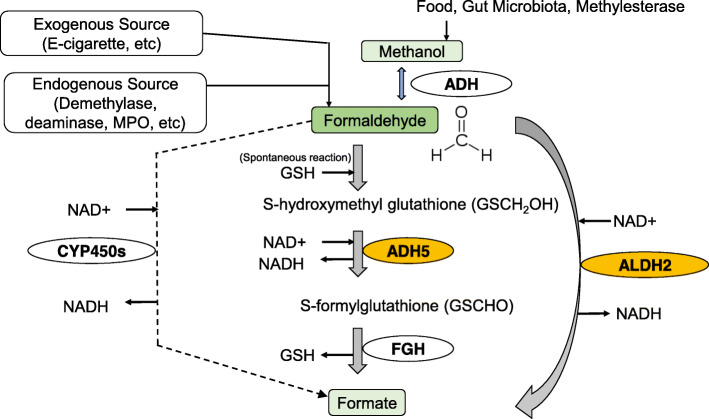
Endogenous formaldehyde metabolism. Endogenous formaldehyde is mainly detoxified via the ADH5 pathway. Formaldehyde is non-enzymatically bound to GSH, oxidized by ADH5, and further metabolized to formic acid by FGH. ALDH2 exists as an enzyme that redundant to the ADH5-dependent detoxification system. The biological significance of oxidation by other formaldehyde detoxification enzymes such as CYP2E1 and ADH appears to be negligible

Various endogenous aldehydes exist in our human body. Among such aldehydes, perhaps the most abundant aldehyde in vivo under physiological conditions is the one-carbon carbonyl compound formaldehyde. It has been reported that formaldehyde is produced in cells by enzymatic reactions, such as oxidative demethylation [1–4]. One function of endogenous formaldehyde is as a single carbon source and a building block to make DNA, RNA and proteins. On the other hand, exogenous formaldehyde is classified by the IARC as a known human carcinogen. The carbonyl group of formaldehyde reacts with amino moieties of DNA and proteins, causing genotoxicity and impaired protein function. For the last two decades, our research interest has been focused on endogenous formaldehyde, and we have proposed that endogenous formaldehyde is a causative agent of non-infectious inflammation, including atherosclerosis [3], and plays an important role in the human hereditary disease Fanconi anemia [5, 6]. Regarding the genotoxicity of formaldehyde, we first demonstrated that chicken DT40 B-lymphocytic cells deficient in the FANC/BRCA pathways are sensitive to physiological levels of formaldehyde (LC50: ~ 5 μM) and the 2-carbon carbonyl compound acetaldehyde at fairly high concentrations (LC50: ~ 2500 μM) [5, 7]. Intracellular formaldehyde is mainly detoxified by cytosolic alcohol dehydrogenase 5 (ADH5, Km = 0.12–6.5 μM) (Fig. 1) [8–11]. However, it has been reported that chicken DT40 cells lacking *ADH5* can grow normally, and their sensitivity to exogenous formaldehyde is not different from wild-type cells [12]. In addition, *Adh5*^*−/−*^ mice developed by the Stamler group are born and develop normally in both sexes [13]. The long-term survival rate of *Adh5*^*−/−*^ mice was also almost the same as that of *wild-type* mice [14]. These results suggest that there exist formaldehyde metabolism pathways that act as backup mechanisms for the ADH5 enzyme. Enzymes other than ADH5 related to the detoxification of formaldehyde include (1) the cytosolic alcohol dehydrogenase (ADH1, Km = 30,000 μM) (reduction) [15]; (2) the mitochondrial aldehyde dehydrogenase 2 (ALDH2, Km = 170–400 μM) (oxidation) [16, 17]; and (3) cytochrome P450 2E1 (CYP2E1) (Km = 1100 μM) (oxidation) [18]. Among them, ALDH2 with a relatively low Km value is considered to be the major compensatory enzyme for ADH5 (Fig. 1).

As with formaldehyde, the two-carbon carbonyl compound acetaldehyde also exists as an endogenous aldehyde. However, acetaldehyde is more than100 times less reactive and less toxic than formaldehyde [5]. Acetaldehyde is primarily metabolized by the mitochondrial ALDH2 (Km < 1 μM) [19]. Similar to ADH5 deficiency, DT40 cells deficient in ALDH2 can grow normally and are as sensitive to acetaldehyde as wild-type cells [12]. Acetaldehyde metabolism is also backed up by a combination of the following enzymes: ALDH1B1 (Km = 30 μM), ALDH1A1 (Km = 50–180 μM) [19], ALDH9A1 (Km = 40–50 μM), and perhaps ALDH1A2 (Km = 650 μM) [19–21]. These compensatory pathways may explain why ALDH2-deficient mice and individuals are born normally and do not exhibit any overt health issues. In this study, therefore, we investigated the impact of the deletion of both the major and compensatory pathways of formaldehyde metabolism (ADH5 and ALDH2) in DNA repair-proficient mice.

## Materials and methods

### Mouse husbandry and mouse genetics

All mouse experiments were approved by the Institutional Animal Care and Use Committees review board at the University of North Carolina at Chapel Hill and were performed in accordance with federal guidelines. Mice were housed in a pathogen-free, temperature- and light-controlled animal facility under a 12-h light/dark cycle and were provided standard food and water ad libitum. *Aldh2*^*−/−*^ mice in a C57BL/6 background and *Adh5*^*−/−*^ mice in a C57BL/6 background were obtained from Dr. Toru Nyunoya (Lovelace Respiratory Research Institute, USA) [22] and Dr. Jonathan Stamler (Case Western University, USA) [13], respectively. C57BL/6 mice were originally purchased from The Jackson Laboratory and bred in our animal facility. All mice used in the present study were in a C57BL/6 background. *Adh5*^*−/−*^ mice were bred using Alpha Dri bedding due to their susceptibility to dermatitis. We attempted to establish *Aldh2*^*−/−*^*/Adh5*^*−/−*^ mice by crossing *Aldh2*^*−/−*^*/Adh5*^*+/−*^ mice.

### Behavior and organ weight

Behavior of *Aldh2*^*−/−*^*/Adh5*^*−/−*^ mice and their heterozygous counterparts in the mouse cage was recorded by video and photograph immediately before euthanasia. At post-natal day 25, *Aldh2*^*−/−*^*/Adh5*^*−/−*^ mice and their *Aldh2*^*−/−*^*/Adh5*^*+/−*^ mice were euthanized by CO_2_ euthanasia. After weighing, blood was collected from the abdominal vein. Livers, spleens, kidneys, brains, lungs, hearts, and thymus were collected and organ weights were measured.

## Results and discussions

**Fig. 2:**
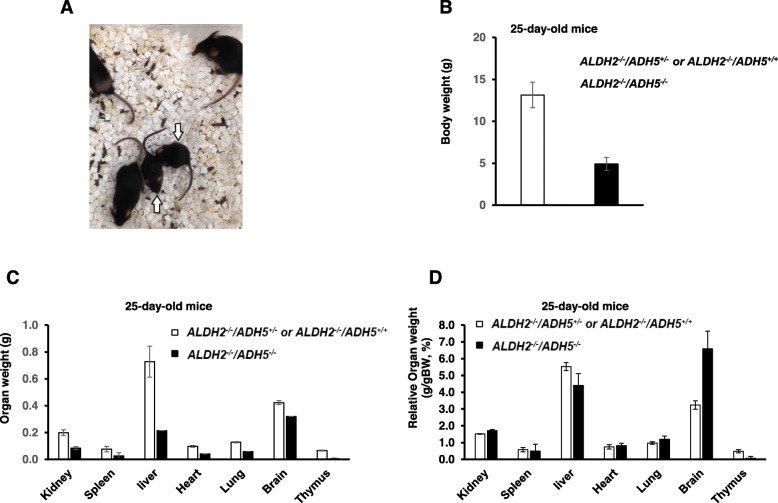
General appearance, body weight and organ weight of Aldh2−/−/Adh5−/− mice. **a** General appearance of 25-day-old Aldh2−/−/Adh5−/− mice (white arrow) and Aldh2−/−/Adh5+/− and Aldh2−/−/Adh5+/+mice in a cage. **b** Body weight (mean ± SD) of Aldh2−/−/Adh5−/− mice (*n* = 2) and Aldh2−/−/Adh5+/− and Aldh2−/−/Adh5+/+ mice (*n* = 3). **c**, **d** Organ weight and relative organ weight (mean ± SD) of Aldh2−/−/Adh5−/− mice (n = 2) and Aldh2−/−/Adh5+/− and Aldh2−/−/Adh5+/+ mice (*n* = 3)


We attempted to generate C57BL/6 mice deficient in both *Adh5* and *Aldh2* genes in order to examine the effects of endogenous formaldehyde in mice with normal DNA repair function. We interbred mice to obtain *Aldh2*^*−/−*^*/Adh5*^*−/−*^ mice in a C57BL/6 background by crossing *Aldh2*^*−/−*^*/Adh5*^*+/−*^ mice. Almost no *Aldh2*^*−/−*^*/Adh5*^*−/−*^ survived past post-natal day 21. The number of mice in the litter occasionally decreased within a few days of birth, suggesting early post-natal lethality in some *Aldh2*^*−/−*^*/Adh5*^*−/−*^ mice. Although the reason is unclear, in one very rare case, *Aldh2*^*−/−*^*/Adh5*^*−/−*^ mice survived up to 25 days after birth. Two of the five littermates were *Aldh2*^*−/−*^*/Adh5*^*−/−*^ mice (two females), as confirmed by genotyping, and three other animals were *Aldh2*^*−/−*^*/Adh5*^*+/−*^ (male and female) and *Aldh2*^*−/−*^*/Adh5*^*+/+*^ (male) mice (Fig. 2a). *Aldh2*^*−/−*^*/Adh5*^*−/−*^ mice weighed only about 37% of that of *Aldh2*^*−/−*^*/Adh5*^*+/−*^ and *Aldh2*^*−/−*^*/Adh5*^*+/+*^ mice (Figs. 2a and b). There were no significant abnormalities in their behavior (Video 1). Small thymus was observed at necropsy after euthanasia. It may also be related to *Adh5*^*−/−*^ mice having fewer number of CD4 single-positive thymocytes via apoptosis [23]. Absolute organ weights of other organs were decreased in the *Aldh2*^*−/−*^*/Adh5*^*−/−*^ mice, whereas, the relative organ weights were almost the same as those of wild type except for the brain (Fig. 2c and d). Therefore, the decreased absolute organ weights are probably due to the secondary effects caused by weight loss. Based on these results, *Aldh2*^*−/−*^*/Adh5*^*−/−*^ animals seem to exhibit a partial synthetic lethality or a phenotype that is lethal during the pre-weaning period. This lethal outcome may be result of an accumulation of endogenous formaldehyde in mouse fetuses or neonates by inactivating both ADH5 and ALDH2. Although we could not measure formaldehyde-derived DNA adducts in the *Aldh2*^*−/−*^*/Adh5*^*−/−*^ organs in this study, we expect that tissues in *Aldh2*^*−/−*^*/Adh5*^*−/−*^ mice may show a marked increase in formaldehyde-derived DNA adducts compared to single *Aldh2*^*−/−*^ and *Adh5*^*−/−*^ mice as well as wild-type mice.

**Additional file 1:** Video 1


The key point of this study is that the simultaneous disruption of two major metabolic pathways involved in endogenous formaldehyde detoxification leads to partial synthetic lethality during embryonic and early post-natal periods in mice with normal DNA damage repair function. Approximately 50% of East Asians have dominant-negative ALDH2*2 alleles, which are low-function variants of ALDH2, and it has been reported that the ALDH2 function of ALDH2*1/*2 is less than 8% compared to that of wild-type ALDH2*1/*1 [16]. As with *Aldh2*-deficient mice, individuals with ALDH2*2 do not show a severe, fatal phenotype [24]. However, unfavorable effects of ALDH2*2 allele have been reported in terms of the risk of some diseases such as osteoporosis [25] and neurodegenerative diseases [24, 26, 27]. These diseases in individuals with ALDH2*2 are not necessarily related to alcohol consumption but rather may be due to endogenous aldehydes. ALDH activity for 10 μM formaldehyde in hepatic mitochondria from individuals with ALDH2*1/*2 was ~ 30% of that from individuals with functional ALDH2*1/*1 [16]. Therefore, endogenous formaldehyde may be elevated in people with ALDH2*1/*2 and ALDH2*2/*2. Recent studies have reported that aldehydes such as formaldehyde and acetaldehyde are complexed to form 1,4-dihydropyrdine-lysine adducts [3, 6, 28], which is an inflammatory, oxidation-specific epitope that can cause the inhibition of osteogenesis [29, 30]. The increased formation of 1,4-dihydropyrdine-lysine adducts in the bone of individuals with the ALDH2*2 allele could cause worse osteoporosis than individuals with ALDH2*1/*1. Likewise, SAMP8 mice, which are used as a model for Alzheimer’s disease, show increased formaldehyde-producing semicarbazide-sensitive amine oxidase (SSAO) and decreased ADH5 activity in the brain [31]. In addition, *Aldh2*^*−/−*^ mice showed elevated hippocampal formaldehyde levels produced by mitochondrial sarcosine dehydrogenase and impairment in memory [32], suggesting that ALDH2 deficiency causes an accumulation of endogenous formaldehyde which may result in memory loss in mice. As such, individuals with the ALDH2*2 allele may exhibit elevated formaldehyde in the brain, which may explain an association between individuals with the ALDH2*2 allele and increased incidence of neurodegenerative diseases.

Although ADH5 is ubiquitously expressed in various animal and human tissues, expression levels of ADH5 may be widely variable between organs and cell types. Indeed, wild-type C57BL/6 mice showed the greatest ADH5 expression in the liver and kidney, whereas the expression of ADH5 in the bone marrow was reported to be tens of times lower than its expression in the kidney [14]. As with mice, ADH5 expression was lowly detected in lymph nodes, spleen, and bone marrow in humans [33]. Based on this evidence, depending on the organs, ADH5 expression may be quite low and formaldehyde metabolism may be in a persistently reduced state. When ADH5 expression is reduced in certain organs and cell types in individuals with dominant-negative ALDH2*2 alleles, the ability to metabolize endogenous formaldehyde may be significantly reduced, possibly resulting in endogenous formaldehyde accumulation. This may be particularly important when hereditary Fanconi anemia occurs under ALDH2 dysfunction. Specifically, *Aldh2*^*−/−*^*/FancD2*^*−/−*^ mice develop aplastic anemia [34], and the disease severity of Japanese Fanconi anemia patients correlates with the presence of a dominant-negative ALDH2*2 allele [35]. Based on this evidence, acetaldehyde has been recognized as an endogenous source of DNA inter-strand crosslinks. However, as described above, endogenous acetaldehyde is unlikely to excessively accumulate in humans with the ALDH2*2 allele due to the existence of many compensatory pathways for acetaldehyde metabolism. Instead, when an individual with mutated Fanconi anemia genes also carries the dominant-negative ALDH2*2 allele, ADH5 expression in hematopoietic tissue may be down-regulated in hematopoietic tissues, which may lead to more serious bone marrow abnormalities such as leukemia compared to patients with wild-type ALDH2*1/*1 alleles.

**Fig. 3:**
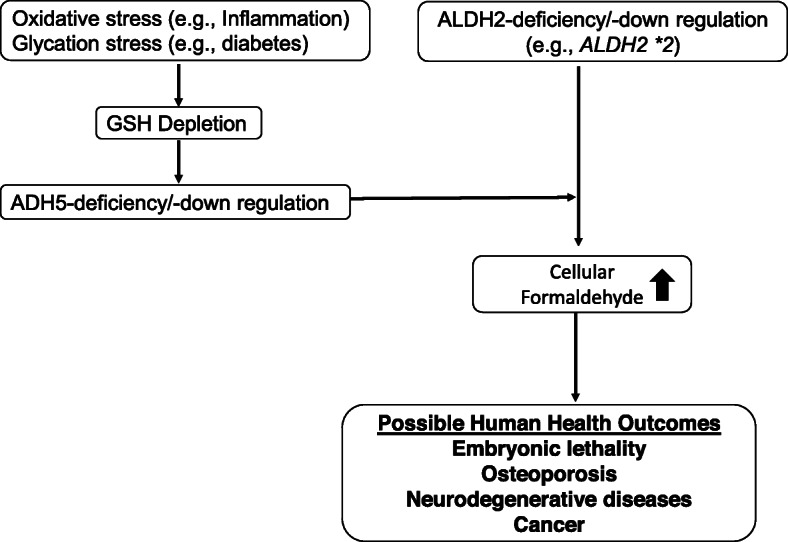
Hypothetical mechanism of possible human diseases caused by endogenous formaldehyde in individuals with the ALDH2*2 allele. GSH depletion occurs due to systemic or local oxidative/glycation stress, leading to the failure of the main formaldehyde detoxification pathway via ADH5. Under such conditions, individuals with the ALDH2*2 allele, which normally oxidizes formaldehyde at lower levels, accumulate intracellular formaldehyde. The increased endogenous formaldehyde may induce various human health problems

In summary, we conclude that more attention should be paid to endogenous aldehydes, especially formaldehyde, in understanding the etiology of diseases that people with ALDH2 dysfunction are susceptible to (Fig. 3). Since more than 50% of the East Asian population has the dominant negative ALDH2*2 allele, it may be that they do not have functional compensatory pathways for endogenous formaldehyde detoxification. In such ALDH2-deficient populations, ADH5 function may be decreased by systemic or local GSH depletion under various pathophysiological conditions, further resulting in endogenous formaldehyde accumulation. Endogenous formaldehyde plays an important role in the pathogenesis of inflammation, osteoporosis, cancer and neurodegenerative diseases. Therefore, it is important to study whether the disease that frequently affects individuals with the ALDH2 * 2 allele is due to high levels of endogenous formaldehyde present in vivo.

## Data Availability

The analyzed dataset and materials during the current study will be provided from the corresponding author on reasonable request.

## Abbreviations

ADH: Alcohol dehydrogenase
ALDH: Aldehyde dehydrogenase
SSAO: Semicarbazide-sensitive amine oxidase

## References

[1] Wu SC, Zhang Y (2010). Active DNA Demethylation: many roads lead to Rome. Nat rev Mol Cell Biol.

[2] Yu PH, Wright S, Fan EH, Lun ZR, Gubisne-Harberle D (2003). Physiological and pathological implications of semicarbazide-sensitive amine oxidase. Biochim Biophys Acta - Proteins Proteomics. Elsevier.

[3] Nakamura J, Shimomoto T, Collins LB, Holley DW, Zhang Z, Barbee JM (2017). Evidence that endogenous formaldehyde produces immunogenic and atherogenic adduct epitopes. Sci Rep.

[4] Burgos-Barragan G, Wit N, Meiser J, Dingler FA, Pietzke M, Mulderrig L (2017). Mammals divert endogenous genotoxic formaldehyde into one-carbon metabolism. Nature.

[5] Ridpath JR, Nakamura A, Tano K, Luke AM, Sonoda E, Arakawa H (2007). Cells deficient in the FANC/BRCA pathway are hypersensitive to plasma levels of formaldehyde. Cancer Res.

[6] Nakamura J, Nakamura M (2020). DNA-protein crosslink formation by endogenous aldehydes and AP sites. DNA Repair (Amst). Elsevier B.V.

[7] Ridpath JR, Nakamura J. Acid-specific formaldehyde donor is a potential, dual targeting cancer chemotherapeutic/chemo preventive drug for FANC/BRCA-mutant cancer. Genes Environ. 2020;41. 10.1186/s41021-019-0136-5 PMC6921423 31890056

[8] Teng S, Beard K, Pourahmad J, Moridani M, Easson E, Poon R, et al. The formaldehyde metabolic detoxification enzyme systems and molecular cytotoxic mechanism in isolated rat hepatocytes. Chem Biol Interact. 2001:285–96. 10.1016/s0009-2797(00)00272-6 11306052

[9] Staab CA, Alander J, Brandt M, Lengqvist J, Morgenstern R, Grafström RC (2008). Reduction of S-nitrosoglutathione by alcohol dehydrogenase 3 is facilitated by substrate alcohols via direct cofactor recycling and leads to GSH-controlled formation of glutathione transferase inhibitors. Biochem J.

[10] Sanghani PC, Stone CL, Ray BD, Pindel EV, Hurley TD, Bosron WF (2000). Kinetic mechanism of human glutathione-dependent formaldehyde dehydrogenase. Biochemistry.

[11] Staab CA, Alander J, Morgenstern R, Grafström RC, Höög J-O (2009). The Janus face of alcohol dehydrogenase 3. Chem Biol Interact.

[12] Rosado IV, Langevin F, Crossan GP, Takata M, Patel KJ (2011). Formaldehyde catabolism is essential in cells deficient for the Fanconi anemia DNA-repair pathway. Nat Struct Mol Biol.

[13] Liu L, Yan Y, Zeng M, Zhang J, Hanes MA, Ahearn G (2004). Essential roles of S-Nitrosothiols in vascular homeostasis and Endotoxic shock. Cell Cell Press.

[14] Pontel LB, Rosado IV, Burgos-Barragan G, Garaycoechea JI, Yu R, Arends MJ (2015). Endogenous Formaldehyde Is a Hematopoietic Stem Cell Genotoxin and Metabolic Carcinogen. Mol Cell.

[15] Skrzydlewska E. Toxicological and metabolic consequences of methanol poisoning. Toxicol Mech Methods. Informa Healthcare; 2003;13:277–293. [cited 2020 mar 12] Available from: http://www.ncbi.nlm.nih.gov/pubmed/20021153. 10.1080/713857189 20021153

[16] Wang R-S, Nakajima T, Kawamoto T, Honma T (2002). Effects of aldehyde dehydrogenase-2 genetic polymorphisms on metabolism of structurally different aldehydes in human liver. Drug Metab Dispos.

[17] Wang M-F, Han C-L, Yin S-J (2009). Substrate specificity of human and yeast aldehyde dehydrogenases. Chem Biol Interact.

[18] Bell-Parikh LC, Guengerich FP (1999). Kinetics of cytochrome P450 2E1-catalyzed oxidation of ethanol to acetic acid via acetaldehyde. J Biol Chem.

[19] Marchitti SA, Brocker C, Stagos D, Vasiliou V. Non-P450 aldehyde oxidizing enzymes: The aldehyde dehydrogenase superfamily. Expert Opin. Drug Metab. Toxicol. 2008:697–720. 10.1517/17425250802102627 PMC2658643 18611112

[20] Uotila L, Koivusalo M (1974). Formaldehyde dehydrogenase from human liver. Purification, properties, and evidence for the formation of glutathione thiol esters by the enzyme. J Biol Chem.

[21] Schmidt RP, Mock RE, Shiner DS (1972). Lactic dehydrogenase in lung tissue and plasma of rhesus monkeys. Lab Anim Sci.

[22] Kitagawa K, Kawamoto T, Kunugita N, Tsukiyama T, Okamoto K, Yoshida A (2000). Aldehyde dehydrogenase (ALDH) 2 associates with oxidation of methoxyacetaldehyde; in vitro analysis with liver subcellular fraction derived from human and Aldh2 gene targeting mouse. FEBS Lett.

[23] Yang Z, Wang Z-E, Doulias P-T, Wei W, Ischiropoulos H, Locksley RM (2010). Lymphocyte development requires S-nitrosoglutathione reductase. J Immunol.

[24] Matsumoto A (2019). The bidirectional effect of defective ALDH2 polymorphism and disease prevention. Adv Exp med Biol [Internet].

[25] Yamaguchi J, Hasegawa Y, Kawasaki M, Masui T, Kanoh T, Ishiguro N (2006). ALDH2 polymorphisms and bone mineral density in an elderly Japanese population. Osteoporos Int.

[26] Kamino K, Nagasaka K, Imagawa M, Yamamoto H, Yoneda H, Ueki A (2000). Deficiency in mitochondrial aldehyde dehydrogenase increases the risk for late-onset Alzheimer’s disease in the Japanese population. Biochem Biophys Res Commun [Internet]. Academic Press Inc.

[27] Wang B, Wang J, Zhou S, Tan S, He X, Yang Z (2008). The association of mitochondrial aldehyde dehydrogenase gene (ALDH2) polymorphism with susceptibility to late-onset Alzheimer’s disease in Chinese. J Neurol Sci.

[28] Shimomoto T, Collins LB, Yi X, Holley DW, Zhang Z, Tian X, et al. A purified MAA-based ELISA is a useful tool for determining anti-MAA antibody titer with high sensitivity. 10.1371/journal.pone.0172172 PMC5319763 28222187

[29] Ambrogini E, Que X, Wang S, Yamaguchi F, Weinstein RS, Tsimikas S (2018). Oxidation-specific epitopes restrain bone formation. Nat Commun.

[30] Rudnick RB, Chen Q, Stea ED, Hartmann A, Papac-Milicevic N, Person F (2018). FHR5 Binds to Laminins, Uses Separate C3b and Surface-Binding Sites, and Activates Complement on Malondialdehyde-Acetaldehyde Surfaces. J Immunol.

[31] Qiang M, Xiao R, Su T, Wu B-B, Tong Z-Q, Liu Y (2014). A novel mechanism for endogenous formaldehyde elevation in SAMP8 mouse. J Alzheimers Dis.

[32] Ai L, Tan T, Tang Y, Yang J, Cui D, Wang R (2019). Endogenous formaldehyde is a memory-related molecule in mice and humans. Commun Biol.

[33] Thul PJ, Lindskog C (2018). The human protein atlas: a spatial map of the human proteome. Protein Sci. Blackwell Publishing Ltd.

[34] Garaycoechea JI, Crossan GP, Langevin F, Daly M, Arends MJ, Patel KJ (2012). Genotoxic consequences of endogenous aldehydes on mouse haematopoietic stem cell function. Nature.

[35] Hira A, Yabe H, Yoshida K, Okuno Y, Shiraishi Y, Chiba K (2013). Variant ALDH2 is associated with accelerated progression of bone marrow failure in Japanese Fanconi anemia patients. Blood.

